# The Association Between Adult Attachment Patterns, Attachment to Group and Mental Health in Israel Following the October 7th Attack: The Role of Emotion Regulation Strategies

**DOI:** 10.3390/ijerph21111443

**Published:** 2024-10-30

**Authors:** Yitshak Alfasi, Avi Besser

**Affiliations:** 1 Department of Behavioral Sciences, Hadassah Academic College, Jerusalem 91010, Israel; 2 Department of Communication Disorders, Hadassah Academic College, Jerusalem 91010, Israel

**Keywords:** adult attachment, attachment to group, emotion regulation strategies, mental health, October 7th attack

## Abstract

We examined the associations between attachment anxiety, attachment avoidance, and attachment to group (to Israel) and mental health scores. The study used a community sample of 434 participants in response to the October 7th attack on Israel. Additionally, we explored the potential mechanisms linking these attachment patterns to mental health scores. We focused on emotion regulation strategies, such as task-focused, emotion-focused, and distance-focused strategies, as potential mediators. The results indicated that the negative direct association between attachment anxiety and mental health is fully mediated by a high emotion-focused strategy and a low task-focused strategy. Attachment avoidance did not have a significant direct association with mental health scores. However, it had significant negative indirect associations through a high emotion-focused strategy and a low task-focused strategy. Attachment to the group (to Israel) showed both direct and indirect positive associations with mental health through a low emotion-focused strategy and a high task-focused strategy. The discussion highlights the role of internal representations of insecure attachment, group attachment, and emotion regulation strategies (especially emotion- and task-focused strategies) in how highly stressful external situations affect mental health.

## 1. Introduction

The Hamas attack on Israel, starting on 7 October 2023, caused widespread devastation and humanitarian challenges across the region, significantly impacting communities and causing collective trauma. A recent study [1], conducted after the October 7th attack, found that Israeli college students experienced moderate to high levels of stress. Key predictors included parenthood, poor sleep quality, living in conflict-affected areas, and increased social media usage. This study builds on these findings to examine resilience and stress factors in Israeli citizens following the October 7th events. Our study specifically investigated the associations between attachment patterns and mental health status among Israeli citizens, along with the mediating role of emotion regulation strategies.

Attachment theory states [2,3,4] that attachment patterns are linked to various emotion regulation strategies for managing stress and anxiety. In addition to romantic attachment patterns, the study explores the influence of group attachment. Smith et al. (1999) suggested that attachment to groups can promote positive emotional responses and serve as a resilience factor similar to secure romantic attachment [5].

### 1.1. The Attachment Behavioral System

Attachment theory [2,3,4] states that humans, like other mammals, are born with behavioral systems essential for survival. These systems include, among others, the sexuality system, the caregiving system, the exploratory system, and the affiliative system. These systems include instinctive, innate, and unlearned behaviors that increase survival chances during evolution [6]. One of these systems is the attachment system, activated in times of danger or threat. This system aims to achieve proximity to the “stronger and wiser” figure, providing security that calms anxiety from perceived threats and serving as a “secure base” for exploration and creativity [7].

During infancy, the primary caregiver acts as the attachment figure to whom the baby turns when feeling threatened or distressed. The caregiver’s availability and responsiveness allow the infant to develop and explore freely, build a stable sense of self-worth, and gain confidence and competence in handling life’s dangers. Conversely, if the caregiver is unavailable or unresponsive, the infant may develop an insecure attachment pattern marked by difficulty coping with stress, emphasizing feelings of helplessness and vulnerability [7]. Using the “Strange Situation” paradigm, Ainsworth et al. [8] identified three main attachment patterns in infancy: the secure pattern, which allows the infant to use the caregiver as a ‘secure base’ during threats, calming down quickly and redirecting mental resources to exploration; the anxious pattern, which is marked by ambivalence between longing for proximity and the fear of abandonment; and the avoidant pattern, characterized by the deactivation of the attachment system and distancing from closeness in response to the caregiver’s unavailability or lack of responsiveness.

Attachment theory states that early relationships with caregivers create mental representations of oneself and others. These representations form the basis for internal working models that affect cognition, emotions, and expectations in future relationships [2,3,4]. The main strategy of the attachment system is to achieve closeness to the primary caregiver. If this strategy fails due to unresponsiveness or unavailability of the caregiver, the baby may adopt one of two strategies: *hyper-activation* or *deactivation*. A hyper-activation strategy means making more attempts to get the caregiver’s attention and being unable to relax, even with the caregiver’s support. Deactivation involves withdrawing from attempts to be close and relying only on oneself to deal with threats or danger [2,3,4,8].

Hazan and Shaver (1987) demonstrated that the same attachment strategies used between infants and caregivers are also activated in romantic relationships [9]. Individuals who hyper-activate the attachment system in romantic relationships typically have high *attachment anxiety*, marked by uncertainty about others’ availability and ongoing worries about rejection and abandonment. People who deactivate the attachment system in romantic relationships typically have high *attachment avoidance*, signified by low emotional involvement in close relationships and a strong reliance on themselves.

### 1.2. Attachment Patterns, Emotion Regulation Strategies, and Reaction to Stressful Events

Bowlby [2,3,4] states that attachment strategies greatly influence how individuals manage stress and anxiety. Lazarus and Folkman’s model explains that stress arises from how individuals evaluate stressful events and their ability to cope. Based on these evaluations, individuals choose either problem-focused coping, which aims to solve the problem, or emotion-focused coping, which reduces emotional distress [10]. Previous research indicates that secure attachment promotes healthy and adaptable emotion regulation. This regulation is defined as the ability to identify, understand, and manage emotions in a manner that allows suitable responses to the situation in accordance with personal and social needs and goals while reducing the intensity of negative emotions and maintaining or enhancing positive emotions [11].

Securely attached individuals effectively use their mental resources to address the root cause of their stress without becoming overwhelmed by anxiety. This balanced emotional response allows them to use their cognitive abilities for constructive problem-solving, consistent with Lazarus and Folkman’s concept of “*problem-focused coping*”, which includes seeking help and making concerted efforts to solve the distressing problem [10]. A strong sense of self-efficacy fosters an optimistic outlook, helping them view challenges as manageable and solvable [12].

In contrast, individuals with high attachment anxiety often magnify threats’ seriousness, express significant neediness and vulnerability, and remain vigilant for signs of distress, using what Lazarus and Folkman termed “*emotion-focused coping*”. Pascuzzo et al. (2013) supported this pattern in a longitudinal study tracking adolescents from age 14 to 22. Their findings indicated that insecure attachment to parents and peers during adolescence predicted anxious romantic attachment in adulthood, mediated by emotion-focused strategies like rumination [13].

Avoidant individuals primarily try to suppress or hide their emotional experiences as part of their deactivation strategy. Experiences with unresponsive attachment figures have taught them that expressing emotions can lead to rejection or punishment [12]. As a result, avoidant individuals see emotional expression as a weakness that clashes with their desire for independence and self-reliance [14]. This causes them to distance themselves from an emotional expression and problem-solving, choosing instead “*distance-focused coping*”, which involves actions to distance from stress sources and prevent stress-related thoughts from activating and triggering negative emotions. These theoretical claims have received strong empirical support; studies on emotion regulation strategies in relationship contexts show that secure attachment is linked to more adaptive coping than insecure attachment. For example, Davis et al. (2003) found that after a romantic relationship ended, attachment anxiety was linked to intense emotional distress and fixation on the lost partner, while avoidant individuals were less likely to seek support and often resorted to alcohol or drugs for coping [15]. Similarly, Sbarra (2006) found that attachment security predicted faster recovery from grief and anger after a breakup and greater acceptance of the situation [16].

Attachment patterns are also connected to emotion regulation strategies in various contexts unrelated to relationships. Research shows that individuals with different attachment patterns use distinct coping mechanisms in stressful situations, significantly impacting their mental health and emotional well-being. For instance, Holmberg et al. (2011) asked participants to describe a recent stressor and rank various emotion regulation strategies from a set of cards, each describing a different approach. The results indicated that higher attachment anxiety is associated with a greater reliance on emotion-focused strategies, while attachment avoidance relates to distancing strategies [17]. In a study by Garrison et al. (2014), participants reported their most significant unpleasant event of the day over seven days. Individuals with high attachment anxiety tended to ruminate, which is an emotion-focused strategy, while those with high attachment avoidance preferred emotional suppression, resulting in lower emotional disclosure—consistent with their tendency to distance themselves from stressors [18].

Several studies have examined how individuals with different attachment patterns use emotion regulation strategies to cope with major life stressors. For example, Berant et al. (2001) conducted a longitudinal study of mothers with infants who have congenital heart disease (CHD). They found that attachment avoidance was linked to poorer mental health and a tendency to view motherhood tasks as more demanding. These mothers were less likely to use problem-solving or support-seeking strategies, choosing instead less adaptive approaches. Similarly, individuals with high attachment anxiety exhibited poor mental health and preferred emotion-focused coping strategies [19]. Likewise, Lopez and Gormley (2002) found that maladaptive emotion-regulation strategies mediated the relationship between attachment anxiety, avoidance, and distress during the college transition [20].

Recent research has shown that emotion regulation strategies are ineffective for individuals with insecure attachment. Messina et al. (2023) studied the use of both intrapersonal strategies (like cognitive reappraisal and suppression) and interpersonal strategies (such as seeking support). They found that individuals with high attachment anxiety often used emotion-focused strategies and sought reassurance, which frequently led to dysregulated emotional states. In contrast, individuals with high attachment avoidance favored maladaptive intrapersonal strategies like suppression to keep emotional distance and avoid seeking support [21]. In a recent study [22], participants viewed negative and neutral images while using different emotion regulation strategies. Results showed that securely attached individuals effectively used cognitive reappraisal to manage their emotions. In contrast, individuals with high attachment anxiety had difficulty regulating arousal and reducing negative emotions. Those with high attachment avoidance experienced increased arousal during attempts to suppress emotions, highlighting the ineffectiveness of distancing strategies [22].

Research shows that different emotion regulation strategies among individuals with various attachment patterns are reflected in brain activity. For example, Lemche et al. (2006) used functional magnetic resonance imaging (fMRI) and found that individuals with high attachment anxiety or avoidance had increased amygdala activity when exposed to stress, indicating heightened emotional arousal [23]. Additionally, Quirin et al. (2010) studied the long-term effects of attachment insecurity on nervous system activity. They found that individuals with high attachment anxiety had fewer cells in the left hippocampus, while those with high attachment avoidance had lower cell concentrations on both sides of the hippocampus [24]. These findings align with existing knowledge, suggesting that high stress levels decrease hippocampal cell concentration, contributing to lower emotion regulation abilities.

Eilert and Buchheim (2023) conducted a meta-analysis that reviewed research on the link between attachment patterns and emotion regulation strategies. They focused on studies examining the neural correlates, highlighting how attachment patterns influence brain activity during emotion processing and regulation [25]. Their findings support the idea that securely attached individuals often use adaptive strategies like cognitive reappraisal, while those with anxious or avoidant attachment tend to rely on maladaptive strategies such as rumination and suppression, which can lead to emotional instability and unresolved negative emotions.

### 1.3. Attachment to Groups

In infancy and early childhood, primary caregivers, often one or both parents, act as attachment figures. As individuals reach adolescence and adulthood, various figures, such as siblings, relatives, close friends, and romantic partners, can serve as attachment figures. Attachment dynamics can also occur with authority figures, such as teachers, managers, therapists, and organizations or social groups [26]. Bowlby (1969) argued that social groups, like individual humans, can serve as attachment figures. He noted that during adolescence and adulthood, attachment behavior is directed not only toward people outside the family but also toward groups and institutions, such as schools, workplaces, religious groups, or political organizations, which can be subordinate or primary attachment figures [2].

From an evolutionary standpoint, belonging to a social group and staying close to it helped our ancestors hunt effectively, protect their homes from intruders, and later thrive in agriculture [27]. The same logic that explains an infant’s need for closeness to a primary caregiver also explains an individual’s need to attach to social groups. Like caregivers, groups protect individuals from danger and provide a secure base for development, creativity, and exploration [28].

In their important work, Smith et al. (1999) proposed that key elements of the attachment system are also important for understanding how individuals connect with social groups [5]. The attachment system includes processes related to emotional dependency, the need for support, and the seeking of support, which are influenced by early experiences and shape expectations and behavior in lifelong relationships. Similarly, mental representations of group membership, formed in early childhood, include views of the group as a source of support and security or as a source of rejection and disappointment [29].

Attachment to a group can be based on interpersonal affection or identification with the group’s identity. Group attachment from interpersonal affection arises from mutual attraction among group members [30], while attachment based on identity depends on how much members identify with the group’s values and identity [31]. Prentice et al. (1994) distinguished between attachment based on a “common bond” and attachment based on a ‘common identity”. Common bond attachment links individuals through emotional ties based on affection, love, and mutual appreciation. In contrast, attachment based on common identity depends on a direct connection to the group’s identity, especially seen in large groups with limited personal connections among members [32].

Seeley et al. (2003) studied gender differences in group attachment types, distinguishing between relational (common bond) and collective (common identity) attachment. Their findings showed that women value groups offering personal emotional connections more, whereas men significantly value collective identity in attachment to groups [33]. Thus, women mainly rely on personal friendships within groups, while men value groups for the collective identity they offer.

Castano and Dechesne (2005) studied how attachment to groups helps manage existential anxiety. Using Terror Management Theory, they suggested that group attachment helps individuals manage this anxiety. Group attachment offers a sense of continuity and immortality beyond personal existence, allowing individuals to feel part of a larger, more enduring social entity [34].

Hogg et al. (2007) found that individuals often feel a stronger attachment to groups during high self-uncertainty. Group attachment offers a sense of belonging and clarity about personal identity, which reduces uncertainty. This effect is stronger when the group is seen as cohesive, meaning it has clear boundaries, internal homogeneity, and a strong sense of unity [35].

DeMarco and Newheiser (2019) studied the link between attachment to a group, group esteem, self-esteem, and investment in the group. They found that individuals with low self-esteem often felt more anxious about their attachment to the group, especially when the group was highly esteemed. In contrast, individuals with high self-esteem tended to avoid attachment to groups that were less esteemed. Group attachment anxiety correlated with higher investment in the group, while avoidance in group attachment related to lower investment [36].

A study of Israeli civilians relocated from Gush Katif during the 2005 Gaza Disengagement found that a stronger sense of belonging to the country predicted lower PTSD symptoms and greater mental well-being. Conversely, feelings of alienation from the country were linked to higher PTSD symptoms and lower well-being [37].

In summary, as Mikulincer and Shaver (2023) describe, groups can provide vital emotional and practical resources for managing threats and challenges [26]. This includes personal fortification (strength-building), a stronger sense of agency and control, and reduced uncertainty. These aspects emphasize the group’s role as a ‘secure base, similar to that of a human attachment figure. Social groups fulfill the attachment role by enhancing feelings of security, which improves self-esteem and emotion regulation. This, in turn, helps people cope better with dangers and threats [26].

### 1.4. Overview and Predictions

The current study aims to examine the relationships between attachment patterns and mental health in Israeli citizens after October 7th, as well as the psychological mechanisms driving these relationships. Research by Besser and Neria emphasizes the important role of insecure attachment, especially attachment anxiety, in influencing psychological responses to terrorism in Israeli civilians. Their 2009 study found that prolonged exposure to terrorism in Southern Israel is linked to higher levels of insecure attachment, perceived stress, and PTSD symptoms, with perceived stress mediating the connection between attachment anxiety and PTSD symptoms. This suggests that insecure attachment may worsen stress responses [38]. The 2010 follow-up study explores how insecure attachment patterns and perceived social support interact, showing that these factors significantly influence the development of PTSD and depressive symptoms in civilians affected by the 2009 Israel–Gaza conflict. Cross-Lagged Panel Correlation (CLPC) path models show that attachment anxiety significantly increases PTSD and depression and affects perceived social support levels [39]. In their 2012 study, the researchers found that low perceived social support mediates the link between attachment anxiety and PTSD symptoms in Israeli evacuees under missile threat, highlighting the importance of interpersonal resources in these contexts [40]. A recent study on predictors of post-traumatic stress symptoms and mental health in Ukrainian civilians during the ongoing Russia-Ukraine war found that low social support and feelings of loneliness are linked to higher depression and PTSD symptoms [41]. Although the study didn’t directly examine attachment patterns, low social support and high loneliness levels are key traits of insecure attachment, especially high attachment anxiety. Similarly, a study of Iraqi civilians exposed to suicide car bombings found a connection between insecure attachment and PTSD symptoms, along with other psychiatric issues [42]. Similarly, a study of social workers in New Orleans after Hurricane Katrina found that insecure attachment predicted more severe post-traumatic stress and secondary trauma symptoms [43]. Together, these studies highlight the importance of addressing insecure attachment to understand mental health symptoms related to trauma in conflict zones.

Previous research (e.g., [18,19]) shows that secure attachment leads to better coping with stress, while high attachment anxiety is linked to more negative effects of stress on mental health. Individuals with high attachment anxiety often use emotion-focused regulation strategies, which can increase their fixation on negative emotions from stress, exaggerate perceived threats, and lower their assessment of available coping resources.

**Hypothesis** **1** **(H1).** *We hypothesize that attachment anxiety will negatively impact mental health in Israeli citizens after the October 7th attack and that this link will be mediated by emotion-focused regulation strategies*.

Findings about avoidant attachment, however, are somewhat inconsistent. On one hand, high avoidant attachment correlates with increased arousal in response to stress [22]. On the other hand, individuals with high avoidant attachment often use defense mechanisms that lower the perceived stress intensity, at least consciously [14].

**Hypothesis** **2** **(H2)—Exploratory.** *Thus, this study will explore whether avoidant attachment relates to mental health in Israeli citizens post-October 7th attack and the role of distance-focused regulation strategies in this connection.*

Finally, this study examines the link between group attachment and mental health in Israeli citizens after the October 7th attack. Theoretical assumptions and past findings suggest that group attachment can act as a resilience factor against stress’s negative effects [26]. This is because it works in a way similar to attachment to human figures. The security it provides supports better coping, effective use of mental resources to tackle stressors, and a positive assessment of available resources for coping.

**Hypothesis** **3** **(H3).** *We hypothesize that group attachment to Israel will have a positive relationship with mental health in Israeli citizens after the October 7th attack and that this relationship will be mediated by problem-focused emotion regulation strategies.*

**Figure 1:**
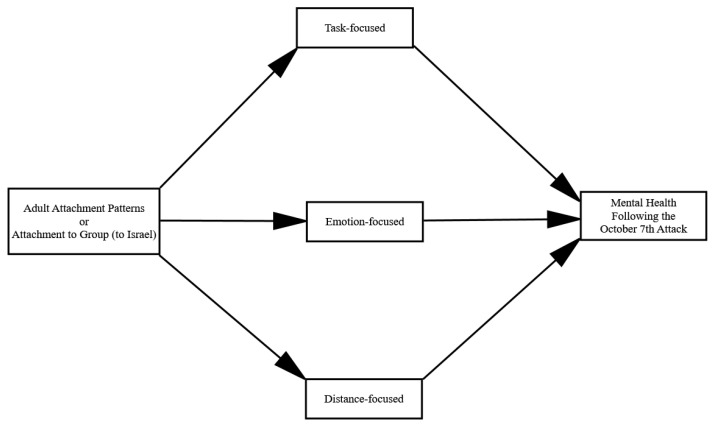
The proposed mediational model in which adult attachment (anxiety and avoidance) and group attachment (to Israel) associations with mental health following the October 7th attack is mediated by emotion-regulation strategies (task, emotion, and distance).

Figure 1 shows the proposed relationships between adult attachment (anxiety and avoidance) and group attachment (to Israel) with mental health after the October 7th attack through emotion-regulation strategies (task, emotion, and distance).

## 2. Materials and Methods

### 2.1. Participants and Procedure

The study used a convenience sample of 434 participants (51% women, 49% men; M age = 32.9, SD = 8.95, Range: 18–50). An a-priori power analysis estimated the required sample size using G*Power 3.1.9 [44]. Based on criteria for large effect size (ES = 0.40) [45], with an alpha = 0.05 and power = 0.95, the projected sample size needed for the current tested models is *n* = 312. Of the participants, 52% identified as secular, 31% as traditional, 14% as religious, and 3% as ultra-orthodox. As to family status, 47.62% defined themselves as singles. Regarding education, 1% had an elementary education, 34% had a secondary education, 16% had non-academic post-secondary education, 33% had a bachelor’s degree, and 16% had a master’s degree or higher. Regarding monthly gross household income, 33% reported earnings below the average, 24% reported similar income, 39% reported earnings above average, and 4% refused to disclose their income level.

The data were collected in November 2023, approximately a month after the 7/10 attack and during the subsequent war between Israel and Hamas. Participants volunteered to participate by responding to recruited efforts through ‘iPanel’, a local online panel of respondents who are obligated to fill out questionnaires. In consequence, the study has no missing data. Participants completed an online survey on a secure website. This included signing online informed consent and completing a demographic questionnaire along with measures of mental health, romantic attachment patterns, attachment to Israel, and emotion regulation strategies. We did not pre-register this study, but the data file is available on the Open Science Framework (OSF) at: https://osf.io/xe6dq/, accessed on 24 October 2024.

### 2.2. Questionnaires

#### 2.2.1. Mental Health

Mental health was measured using the Mental Health Inventory (MHI-5) [46]. This scale assesses mental health status through a five-item screening test that evaluates feelings of anxiety, depression, positive affect, and behavioral/emotional control. Participants were asked to indicate the extent to which they had felt the following way during the last few weeks since the war broke out, using a scale from 1 (very little) to 7 (very much): ‘nervous and tense’ (anxiety), ‘calm and peaceful’ (positive affect), ‘discouraged and depressed’ (depression), ‘happy and joyful’ (positive affect), ‘so down in the dumps that nothing could cheer you up’ (behavioral/emotional control). Items were coded so that higher scores reflect greater mental health. In relation to its psychometric properties, the scale showed validity based on the internal structure: Principal component analysis, with an unrotated factor solution, suggested a single factor (Eigenvalue = 3.285) as the best solution, with a total explained variance of 65.707% [using maximum likelihood (ML) as the extraction method indicated that a single factor solution was adequate to explain the covariances among variables χ^2^ = 52.389, df = 5, *p* < 0.0001. It also presents adequate levels of internal consistency (Cronbach’s α = 0.87).

#### 2.2.2. Romantic Attachment

Romantic attachment patterns were assessed by Wei et al.’s (2007) short form [47] of the Experiences in Close Relationship Scale (ECR-S; [48]), a 12-item self-report adult attachment style questionnaire that measures attachment patterns in adult romantic relationships. The ECR-S demonstrated adequate validity and factor structure [48]. Participants were instructed to think about their prototype experiences in romantic relationships and rate their agreement with each item on a 7-point Likert scale, ranging from 1 (strongly disagree) to 7 (strongly agree). In total, six items assessed Attachment Anxiety (e.g., “I worry that romantic partners won’t care about me as much as I care about them”), and six items assessed Attachment Avoidance (e.g., “I try to avoid getting too close to my partner”; α = 0.78). In relation to its psychometric properties, the scale showed validity based on the internal structure: Principal component analysis, with no rotation, suggested two factors (Eigenvalue = 4.301 and 2.560) as the best solution, with a total explained variance of 57.176% (35.840% and 21.335%, respectively). Using maximum likelihood (ML) as the extraction method indicated that a two-factor solution was adequate to explain the covariances among variables (χ^2^ = 168.516, df = 43, *p* < 0.0001). It also presents adequate levels of internal consistency (α = 0.79 and α = 0.78 for Attachment Anxiety and Avoidance, respectively).

#### 2.2.3. Group Attachment

Group attachment to Israel was assessed using items adapted from Smith et al.’s (1999) Social Group Attachment Scale [5], an 8-item self-report measure. Participants were asked to rate their agreement with eight statements reflecting their attachment to Israel on a 7-point Likert scale, ranging from 1 (strongly disagree) to 7 (strongly agree). For example, “I feel comfortable being dependent on Israel”, “I know Israel will be there when I need it”, and “I find it difficult to trust Israel completely or to be dependent on Israel” (reversed item). In relation to its psychometric properties, the scale showed validity based on the internal structure: Principal component analysis, with an unrotated factor solution, suggested a single factor (Eigenvalue = 4.653) as the best solution, with a total explained variance of 58.158% using maximum likelihood (ML) as the extraction method indicated that a single factor solution was adequate to explain the covariances among variables χ^2^ = 215.541, df = 20, *p* < 0.0001. It also presents adequate levels of internal consistency (Cronbach’s α = 0.89).

#### 2.2.4. Emotion Regulation Strategies

Emotion Regulation Strategies were assessed with the Coping Inventory for Stressful Situations (CISS; [49]), a 21-item self-report questionnaire that evaluates emotion regulation strategies in response to stressful situations. The CISS measures three emotion regulation strategies: Task-focused, emotion-focused, and distance-focused, with each strategy assessed by seven items. Participants were instructed to indicate how much they engage in various types of activities when encountering difficult, stressful, or upsetting situations. Task-focused items refer to problem-solving strategies (e.g., “Focus on the problem and see how I can solve it”), emotion-focused refers to strategies aimed at regulating emotions (“Blame myself for having gotten into this situation”), and distance-focused refers to strategies aimed at avoiding or denying the stressor (“Take some time off and get away from the situation”). In relation to its psychometric properties, the scale showed validity based on the internal structure: Principal component analysis, with no rotation, suggested three factors (Eigenvalue = 4.598, 3.664, 2.473) as the best solution, with a total explained variance of 51.119% (21.897%, 17.447%, and 11.775%, respectively). Using maximum likelihood (ML) as the extraction method indicated that a three-factor solution was adequate to explain the covariances among variables χ^2^ = 414.660, df = 150, *p* < 0.0001). It also presents adequate levels of internal consistencies of all three subscales, as measured by Cronbach’s alpha: Task-focused (α = 0.85), emotion-focused (α = 0.86), and distance-focused (α = 0.75).

### 2.3. Ethics Statement

Participation in this study was voluntary, and participants were aware that they could withdraw from the study at any time. All participants signed and provided online informed consent. No social security numbers or other identifying data were collected, nor were any invasive examinations conducted. This project was conducted with the approval of the Ethics Committee (IRB) of Hadassah Academic College.

### 2.4. Statistical Analysis

First Pearson bivariate correlation tests were performed to test the associations among Attachment Anxiety, Attachment Avoidance, Attachment to Group (to Israel), Emotions, Task, and Distance Emotional Regulation strategies, and Mental Health scores. Data were evaluated using the statistical program SPSS version 26 (SPSS Inc., Chicago, IL, USA). Then we analyzed the direct-indirect effects models (mediational) using Path Analyses with AMOS (Version 29, Arbuckle, 2023) [50] using the maximum-likelihood method. Because of the overlap between the attachment variables, we were concerned that including all of them in the same analysis would make it difficult to understand how they were associated with the mediators and the outcome (see Lynam et al., 2006, for an extended discussion of this “perils of partialling” issue [51]). This prompted us to conduct separate conditional process analyses such that each personality trait served as the predictor in its own model. We performed all statistical tests using two-tailed tests of significance and confidence intervals based on the level of *p* < 0.05.

## 3. Results

### 3.1. Univariate Analyses

Table 1 shows the correlation coefficients and descriptive statistics. The values of Skewness and Kurtosis indicate that the data are fairly symmetrical [52,53,54]. Attachment Anxiety had positive correlations with the Emotion-focused strategy and negative correlations with Mental Health scores. Attachment Avoidance showed positive correlations with the Emotion-focused strategy but was not correlated with Mental Health scores. Attachment to the Group (to Israel) showed a negative correlation with the Emotion-focused strategy and a positive correlation with Mental Health scores. The Emotion-focused strategy had a negative correlation with Mental Health scores, while the Distance-focused strategy was not correlated with them. Attachment Anxiety and Avoidance had low negative correlations with Attachment to the Group (to Israel), indicating that although correlated, these are relatively distinct constructs (*r* = −0.19 and −0.15, *p* < 0.001, respectively).

We examined if there was multicollinearity among the study variables. Eigenvalues of the scaled and uncentered cross-products matrix, condition indices, variance decomposition proportions, variance inflation factors (VIF), and tolerances from the multicollinearity analyses indicated no multicollinearity. To assess the potential impact of common method bias, we conducted Harman’s one-factor test using all items from the four questionnaires in our analysis. This method examines the variance explained by one factor using Principal Axis Factoring as the extraction method. The unrotated factor solution indicated that the total variance extracted by one factor was 15.619%, well below the accepted threshold of 50% [55]. Therefore, we conclude that common method bias is not likely to be a significant issue in this study.

### 3.2. Multivariate Analyses

We performed three direct and indirect effects path mediation analyses, controlling for the shared variance among the mediators. In the first analysis, Attachment Anxiety was the predictor; in the second, it was Attachment Avoidance; and in the third, it was Attachment to Group (to Israel). The results of the path mediation analyses are shown in Table 2, Table 3 and Table 4.

**Figure 2:**
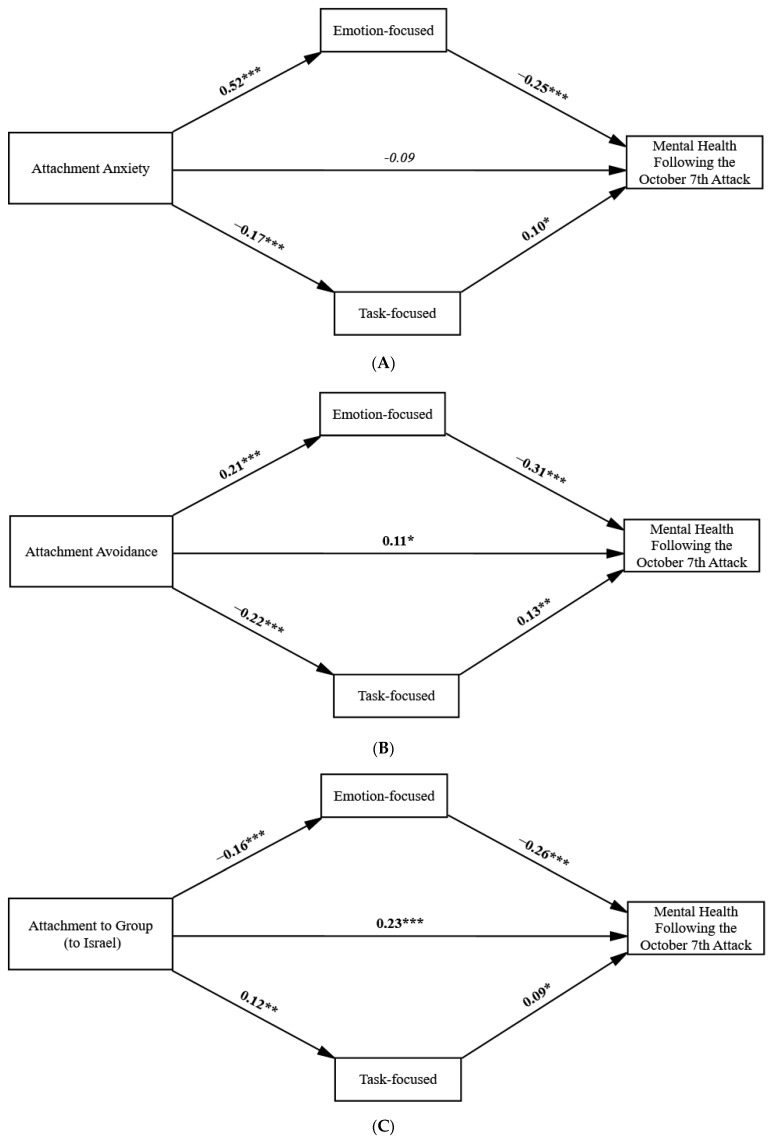
(**A**–**C**) The final direct and indirect effect models. * *p* < 0.05; ** *p* < 0.01; *** *p* < 0.001.

We first evaluated how sociodemographic variables (gender, age, family status, religiosity, years of formal education, and monthly income) affect mental health scores, the study’s outcome variable. We found no significant links between participants’ age, years of education, or monthly income and mental health scores. Gender had a significant impact on mental health (*t*_[432]_ = 8.48, *p* < 0.001), with men reporting higher scores than women. Married individuals had higher mental health scores compared to singles (*t*_[432]_ = −2.39, *p* < 0.05), and higher religiosity levels were also linked to better mental health (*r* = 0.29, *p* < 0.001). Accordingly, in preliminary analyses of the models in Figure 2A–C, we controlled for the effects of these sociodemographic variables (i.e., gender, family status, and religiosity) on the predictors, mediators, and outcome variables. However, these variables did not significantly affect the findings. Consequently, sociodemographic variables were excluded from the final analyses, and differences based on these variables are not discussed to keep the text clear and concise.

#### 3.2.1. Attachment Anxiety and Mental Health

Table 1 shows that the direct total association between attachment anxiety and mental health scores (β = −0.23, *t* = −4.96, CI_95%_ [−0.333, −0.132], *p* < 0.0001) decreased when mediators were included (β = −0.08, *t* = −1.57, ns). Table 2 shows that attachment anxiety is linked to a high emotion-focused strategy, which in turn is linked to low mental health scores. Additionally, attachment anxiety is associated with low task-focused strategy, which also relates to low mental health scores. No significant indirect associations were found for the distance-focused emotion regulation strategy.

#### 3.2.2. Attachment Avoidance and Mental Health

Although there was no significant total effect of attachment avoidance on mental health scores (as shown in Table 1), we explored potential indirect associations through the mediators. Table 3 shows that attachment avoidance is linked to each of the mediators (positively with emotion and negatively with task- and distance-focused emotion regulation strategies). Additionally, attachment avoidance is indirectly linked to mental health scores through emotion and task-focused emotion regulation strategies. No significant indirect associations were found for the distance-focused emotion regulation strategy. Table 3 shows that the nearly zero and nonsignificant total effect of attachment avoidance on mental health scores (β = 0.01, *t* = 0.30, ns) increased and became significant (β = 0.11, *t* = 0.207, *p* < 0.04) when mediators were included. This trend is referred to as inconsistent mediation, which occurs when multiple mediator models show different signs of mediated effects due to opposing indirect associations [56].

### 3.3. Attachment to Group (to Israel)

The total association between Attachment to Group (to Israel) and Mental Health scores (*β* = 0.29, t = 6.21, CI_95%_ [0.181, 0.375], *p* < 0.0001) remained significant even with the mediators included *(β* = 0.23, t = 5.20, *p* < 0.0001). Table 4 shows that attachment to a group (to Israel) is also indirectly linked to mental health through a low emotion-focused strategy, which in turn relates to high Mental Health scores. Attachment to the group (to Israel) is linked to a high emotion-focused strategy, which is associated with low mental health scores. Additionally, attachment anxiety is linked to low task-focused strategy, which is associated with low mental health scores. No significant indirect associations were found for the distance-focused emotion regulation strategy. Attachment to Group (to Israel) is also linked to mental health scores through high task-focused emotion regulation scores, which are associated with high mental health scores.

In summary, attachment anxiety’s negative direct association with mental health is fully mediated by high emotion-focused and low task-focused strategies. Attachment Avoidance shows no significant association with mental health scores. However, it has a significant negative indirect association through high emotion-focused and low task-focused strategies. Attachment to a group (to Israel) has both direct and indirect positive associations with mental health through low emotion-focused and high task-focused strategies. Path coefficients were estimated using 5000 bootstrap samples. All bootstrap samples (100%) converged. Table 2, Table 3 and Table 4 shows that the 95% confidence intervals and the percentile bootstrap confidence intervals for the estimated parameters and indirect effects support the conclusion that the indirect effects are significantly different from zero. The results indicate that the procedure provided a stable estimate of the distributions.

To create the most parsimonious models, we removed the distance-focused strategy and reanalyzed the models. The final models are shown in Figure 2A–C. The indirect effects of an emotion-focused regulation strategy are stronger than those of a task-focused regulation strategy.

## 4. Discussion

Our study found a significant negative association between attachment anxiety and mental health. This association was fully mediated by high emotion-focused coping and low task-focused regulation strategies. While attachment avoidance had no direct effect on mental health, it showed significant indirect effects through high emotion-focused regulation and low task-focused strategies. Interestingly, attachment to a group—specifically attachment to Israel—showed a positive association with mental health. This positive association was seen both directly and indirectly through a low emotion-focused strategy and a high task-focused strategy.

Our findings are consistent with previous research that emphasizes the vulnerability of individuals with insecure attachment patterns to the negative effects of stress in general [1,2,3], especially in their psychological responses to terrorist attacks among Israeli civilians [38,39,40]. Our results support the idea that individuals with high attachment anxiety are more likely to use emotion-focused regulation strategies, which may worsen their distress [4,6]. This mediation effect highlights the critical role of emotion regulation in the complex relationship between insecure attachment and mental health, as it involves both emotion-focused and task-focused regulation strategies [2,7,26]. Our findings suggest that individuals with high attachment anxiety may struggle to allocate mental resources for problem-solving, possibly relying on less adaptive emotion-focused strategies, leading to poorer mental health outcomes [3,8].

The lack of a direct effect of attachment avoidance on mental health, along with significant indirect effects through emotion regulation strategies, highlights the complex relationship between attachment avoidance and mental well-being [9,14]. While individuals with high attachment avoidance may seem less distressed at first, they might be using maladaptive emotion regulation strategies like suppression, which can result in unresolved negative emotions and ultimately harm their mental health [10,12,13]. These mediation effects of both emotion-focused and task-focused strategies highlight the need to recognize the complexities of avoidant coping mechanisms. Even though avoidant individuals may try to minimize emotional expression, they can still be vulnerable to the negative effects of stress because they rely on less adaptive emotion regulation strategies.

Both adult attachment dimensions are linked to greater use of emotion-focused regulation strategies, which are associated with lower levels of mental health. This finding is more pronounced for attachment anxiety, which relies more heavily on emotion-oriented strategies. Additionally, both attachment anxiety and avoidance were significantly linked to lower use of task-focused strategies, contributing to poorer mental health. The indirect effect of insecure attachment on mental health is weaker than the effect of intense use of emotion-focused strategies. Unlike adult attachment, attachment to the group (Israel) shows the opposite association with emotional regulation strategies, demonstrating significantly lower use of emotion-focused and higher use of task-focused strategies, which enhance mental health. Attachment to the group (Israel) may reflect a secure-like internal representation that enhances effective regulation during national traumatic events.

Our analysis found that distance-focused strategies—those involving emotional detachment or cognitive distancing from stressors—did not significantly mediate the relationship between attachment patterns, group attachment, and mental health outcomes. One possible explanation is that individuals with strong group attachment or secure attachment patterns often use engagement strategies, such as seeking support and cognitive reappraisal, instead of distancing themselves. Engagement strategies are frequently associated with better mental health outcomes. Furthermore, distance-focused strategies may not have a significant impact because they are typically less effective at promoting long-term mental health benefits than engagement strategies, which actively manage emotional responses. This suggests that while distance strategies may offer temporary relief, they do not lead to significant improvements in mental health scores for individuals with various attachment patterns. Over the years, research on attachment patterns and reactions to stressful events has mainly focused on personal stressors (e.g., [17,18]). This study adds to the existing knowledge by showing that collective stressors, such as wars, natural disasters, and pandemics, can uniquely challenge the coping patterns of individuals with different attachment styles. This study builds on evidence gathered during the global COVID-19 pandemic, which highlighted the link between attachment patterns and psychological coping during the pandemic. Important findings have come from several countries, including Italy [57], Australia [58], New Zealand [59], and China [60]. Research conducted in Israel during two waves of the COVID-19 pandemic [61] is particularly relevant, finding a link between high attachment anxiety and avoidance and high-stress levels.

Another significant finding of this study is the positive relationship between attachment to a group (in this case, Israel) and mental health. These findings support prior research that suggests group attachment provides security and support during stressful times, effectively reducing trauma’s negative effects [5,31,32]. This finding is especially important in the context of the October 7th attack, where participants faced significant threats and uncertainties. The indirect effects observed through low emotion-focused and high task-focused regulation strategies highlight the importance of understanding how group attachment affects mental health. Our findings suggest that individuals with a strong attachment to a group are more likely to use adaptive emotion regulation strategies, resulting in better mental health outcomes [33,34,35].

These findings are especially intriguing because a key characteristic of the October 7th attack was a profound sense of abandonment. For hours, civilians had to defend themselves against horrific terrorist acts without state security forces, particularly the Israel Defense Forces (IDF), which has a unique role in Israeli society due to mandatory military service, known as “the people’s army”. In the citizen-state relationship, this experience resembles parental abandonment during danger; those meant to protect and save deserted their children. The events of October 7th echoed collective trauma for the Jewish people. The State of Israel was created to offer Jews refuge and a “safe haven”. The rupture is significant after the October 7th attack, as citizens called for help from a state meant to ensure such events would never occur again, like during the Holocaust, but there was no response.

Given this, the study participants’ ability to see their attachment to Israel as a source of psychological resilience, helping them cope with the trauma from the October 7th attack—even in a survey conducted weeks after the events—offers optimism about Israeli society’s mental resilience. It also highlights the importance of identity and belonging, which are central to our psychological immune system. A stable and secure emotional attachment to a group improves the ability to cope with traumatic events, both collectively and individually, as demonstrated on October 7th.

The World Happiness Report (WHR; [62]), produced by the Oxford Wellbeing Research Centre and the UN Sustainable Development Solutions Network, ranks Israel as one of the happiest countries in the world. In the latest ranking, released in March 2024 and covering 2021–2023, Israel ranked 5th, ahead of the United States, the United Kingdom, and most Western European countries. The WHR measures happiness levels among countries using a mix of subjective and objective indicators. The main measure uses survey data where respondents rate their life satisfaction on a scale from 0 to 10. In Israel, a representative sample of about 3000 participants reported an average life satisfaction score of 7.47. However, a survey conducted at the end of 2023, just after the October 7th attack, showed a significant drop, with the average falling to 6.78, placing Israel in 19th place. This contrasts with an average of 7.61 from 2021 to 2022, which would have ranked Israel 2nd.

Israel’s high WHR ranking is due to relative economic prosperity (ranked 27th in GDP per capita) and high life expectancy (5th place), but mainly reflects strong values of family and community (9th in social support). When asked, “If you were in trouble, do you have relatives or friends you can count on for help?”, 94.1% of Israelis answered positively. In Israel, family and interpersonal relationships are very important. This is shown in high fertility rates, with an average of 2.9 children per woman. Even secular women have an average of 2.3 children, which is much higher than the OECD average of 1.6. The value placed on family, friends, and community, along with a sense of life meaning, may explain why, despite challenges, people in Israel feel more satisfied with their lives than in most other countries.

### 4.1. Clinical Implications

Our findings have important implications for mental health professionals who work with individuals experiencing stress, trauma, and conflict. Understanding a person’s attachment pattern is essential. Attachment assessments can help identify individuals with insecure attachment styles, like high attachment anxiety or avoidance. This information can guide the creation of targeted interventions that address vulnerabilities related to attachment and promote adaptive coping skills.

Therapeutic approaches should emphasize emotion regulation strategies, especially for individuals with insecure attachment patterns. Teaching strategies such as problem-solving, relaxation techniques, cognitive reappraisal, and acceptance are helpful. It’s also essential to help individuals recognize and adjust maladaptive strategies like rumination or suppression. Our findings indicate that emotion-focused strategies may be less effective, emphasizing the need for adaptive, cognitive-based approaches.

Encouraging a sense of belonging and support in therapeutic groups is important. Creating connections between individuals with shared experiences or values can greatly support recovery. Group interventions can enhance resilience, improve coping skills, and create community by providing a safe space for sharing experiences and learning from others. This group support is especially helpful for individuals who feel isolated during trauma or conflict.

Professionals should differentiate between individual and group attachment models in their practice. Although individual attachment practices are vital for personal emotion regulation, group attachment improves communal well-being and emotional health.

Finally, mental health professionals should adopt a trauma-informed approach that considers attachment, emotion regulation, and trauma history. Engaging with social support networks and community resources is essential. Integrating current research and evidence-based interventions tailored to client needs, along with collaboration with researchers to improve clinical practice, can foster Post-Traumatic Growth (PTG; [63]). PTG involves positive changes like improved relationships and a renewed appreciation for life, supported by social support, resilience, and cognitive processing. Addressing collective trauma, like the October 7th attack in Israel, can strengthen collective resilience and reinforce social support systems.

### 4.2. Limitations and Future Directions

The cross-sectional design of our study limits our ability to establish causal relationships among attachment, emotion regulation, and mental health. Longitudinal research is necessary to establish temporal precedence and fully understand how these factors develop over time. Relying on self-reported measures exposes the study to biases such as social desirability and recall bias. Future research could improve by using objective measures or incorporating multiple methods for a more comprehensive assessment of these constructs. Using path analysis without including latent variables, which could address measurement error, prevents calculating fit indices and creates uncertainty about the model-data fit. Future research could improve by using structural equation modeling (SEM) with latent variables. This method would better address measurement errors and provide fit indices, enhancing the robustness and clarity of the findings. The sample included only Israeli citizens, limiting the generalizability of our findings to other populations. More research is needed to explore these relationships in different cultural contexts to assess the universality or cultural specificity of the observed associations.

Future studies should further explore the role of other emotion regulation strategies, like reappraisal and acceptance, in relation to attachment and mental health. Exploring how these strategies interact could show their potential to enhance well-being during stressful times. Longitudinal studies are essential to follow individuals’ experiences over time, especially during ongoing conflict or trauma, to better understand the complex relationship between attachment, emotion regulation, and mental health. In-depth research is needed to explore the complexities of group attachment, including different types (e.g., common identity vs. common bond), group cohesion, leadership styles, and group identity, and how these factors affect mental well-being in times of crisis. Cross-cultural research is necessary to assess the universality or cultural specificity of the associations between attachment, emotion regulation, and mental health. Comparing findings from various cultural groups could reveal potential cultural influences on these associations.

## 5. Conclusions

Despite its limitations, our study emphasizes the important role of attachment patterns, emotion regulation strategies, and group attachment in understanding mental health during sudden and extreme collective stress. The findings highlight the need for mental health professionals to consider these factors when assessing and treating individuals experiencing trauma and stress. More research is needed to improve our understanding of these relationships and to create effective interventions that promote resilience and well-being.

## Figures and Tables

**Table 1:** Intercorrelations and descriptive statistics.

	1	2	3	4	5	6	7
1. Attachment Anxiety	–						
2. Attachment Avoidance	0.34 ***	–					
3. Attachment to Group (to Israel)	−0.19 ***	−0.15 **	–				
4. Emotion-focused	0.52 ***	0.21 ***	−0.16 ***	–			
5. Task-focused	−0.17 ***	−0.22 ***	0.13 **	−0.14 **	–		
6. Distance-focused	0.06	−0.26 ***	0.00	0.08	0.17 ***	–	
7. Mental Health	−0.23 ***	0.01	0.29 ***	−0.31 ***	0.15 ***	−0.06	
*Mean*	3.43	2.58	4.26	3.73	4.73	4.14	3.06
*Standard Deviation*	1.26	1.06	1.31	1.30	1.08	1.13	1.28
*Skewness*	0.17	0.32	0.14	0.30	−0.39	−0.00	0.72
*Kurtosis*	−0.33	−0.68	−0.53	−0.33	0.49	−0.36	0.28

** *p* < 0.01; *** *p* < 0.001.

**Table 2:** Results of the path mediation analyses for attachment anxiety.

	Estimates				Bootstrap					
								PC Confidence		
Effect	β	SE	*t*	*p*<	SE	Bias	SE-Bias	Lower	Upper	*p*
***Associations with Mediators***										
Attachment Anxiety → Emotion-focused	0.52	0.042	12.62	0.0001	0.046	0.001	0.001	0.444	0.624	0.0001
Attachment Anxiety → Task-focused	−0.17	0.041	−3.48	0.0001	0.051	0.001	0.001	−0.237	−0.040	0.007
Attachment Anxiety → Distance-focused	0.06	0.043	1.33	ns	0.046	0.000	0.001	−0.033	0.147	0.205
***Associations with Outcome***										
Attachment Anxiety → Mental Health (Direct)	−0.08	0.054	−1.57	ns	0.055	0.002	0.001	−0.190	0.027	0.138
Emotion-focused → Mental Health	−0.24	0.052	−4.56	0.0001	0.055	−0.002	0.001	−0.344	−0.130	0.0001
Task-focused → Mental Health	0.12	0.055	2.45	0.02	0.060	−0.002	0.001	0.019	0.254	0.025
Distance-focused→ Mental Health	−0.05	0.052	0.24	ns	0.055	−0.002	0.001	−0.172	0.042	0.255
***Indirect effect***					0.032			−0.215	−0.091	0.0001

Based on 5000 bootstrap samples. PC confidence = Percentile Confidence Intervals (95%).

**Table 3:** Results of the path mediation analyses for attachment avoidance.

	Estimates				Bootstrap					
								PC Confidence		
Effect	β	SE	*t*	*p*<	SE	Bias	SE-Bias	Lower	Upper	*p*
***Associations with Mediators***										
Attachment Avoidance → Emotion-focused	0.21	0.058	4.46	0.0001	0.061	0.001	0.001	0.137	0.380	0.0001
Attachment Avoidance → Task-focused	−0.22	0.048	−4.71	0.0001	0.048	0.000	0.001	−0.310	−0.129	0.0001
Attachment Avoidance → Distance-focused	−0.26	0.050	−5.52	0.0001	0.051	−0.001	0.001	−0.375	−0.177	0.0001
***Associations with Outcome***										
Attachment Avoidance → Mental Health (Direct)	0.11	0.059	2.07	0.04	0.063	0.000	0.001	0.001	0.245	0.049
Emotion-focused → Mental Health	−0.31	0.046	−6.50	0.0001	0.052	−0.001	0.001	−0.400	−0.197	0.0001
Task-focused → Mental Health	0.14	0.056	2.95	0.01	0.063	−0.001	0.001	0.040	0.287	0.010
Distance-focused→ Mental Health	−0.03	0.054	−0.67	ns	0.057	−0.001	0.001	−0.151	0.073	0.511
***Indirect effect***					0.036			−0.178	−0.038	0.001

Based on 5000 bootstrap samples. PC confidence = Percentile Confidence Intervals (95%).

**Table 4:** Results of the path mediation analyses for attachment to group (to Israel).

	Estimates				Bootstrap					
								PC Confidence		
Effect	β	SE	*t*	*p*<	SE	Bias	SE-Bias	Lower	Upper	*p*
***Associations with Mediators***										
Attachment to Group (to Israel) → Emotion-focused	−0.16	0.047	−3.46	0.0001	0.049	0.000	0.001	−0.256	−0.066	0.001
Attachment to Group (to Israel) → Task-focused	0.13	0.039	2.61	0.01	0.039	−0.001	0.001	0.025	0.180	0.009
Attachment to Group (to Israel) → Distance-focused	0.00	0.042	0.17	ns	0.043	−0.001	0.001	−0.079	0.091	0.88
***Associations with Outcome***										
Attachment to Group (to Israel) → Mental Health (Direct)	0.23	0.044	5.20	0.0001	0.046	0.000	0.001	0.136	0.318	0.0001
Emotion-focused → Mental Health	−0.25	0.044	−5.51	0.0001	0.048	−0.001	0.001	−0.337	−0.150	0.0001
Task-focused → Mental Health	0.10	0.054	2.18	0.03	0.059	−0.002	0.001	0.001	0.231	0.048
Distance-focused → Mental Health	−0.06	0.051	0.20	ns	0.054	−0.001	0.001	−0.173	0.036	0.219
***Indirect effect***					0.017			0.020	0.088	0.0001

Based on 5000 bootstrap samples. PC confidence = Percentile Confidence Intervals (95%).

## Data Availability

The data presented in this study are available on request from the corresponding authors.

## References

[1] Dopelt K., Houminer-Klepar N. (2024). War-Related Stress among Israeli College Students Following 7 October 2023 Terror Attack in Israel. Eur. J. Investig. Health Psychol. Educ..

[2] Bowlby J. (1969). Attachment and Loss: Vol. 1. Attachment.

[3] Bowlby J. (1973). Attachment and Loss: Vol. 2. Separation: Anxiety and Anger.

[4] Bowlby J. (1980). Attachment and Loss: Vol. 3. Sadness and Depression.

[5] Smith E.R., Murphy J., Coats S. (1999). Attachment to groups: Theory and management. J. Personal. Soc. Psychol..

[6] Mikulincer M., Shaver P.R. (2016). Attachment in Adulthood: Structure, Dynamics, and Change.

[7] Bowlby J. (1988). A Secure Base.

[8] Ainsworth M.D.S., Blehar M.C., Waters E., Wall S.N. (1978). Patterns of Attachment: A Psychological Study of the Strange Situation.

[9] Hazan C., Shaver P.R. (1987). Romantic love conceptualized as an attachment process. J. Personal. Soc. Psychol..

[10] Lazarus R.S., Folkman S. (1984). Stress, Appraisal, and Coping.

[11] Shaver P.R., Mikulincer M., Gross J.J. (2007). Adult attachment strategies and the regulation of emotion. Handbook of Emotion Regulation.

[12] Cassidy J., Fox N.A., Campos J.J. (1994). Emotion regulation: Influence of attachment relationships. The Development of Emotion Regulation: Biological and Behavioral Considerations. Monographs of the Society for Research in Child Development.

[13] Pascuzzo K., Cyr C., Moss E. (2013). Longitudinal association between adolescent attachment, adult romantic attachment, and emotion regulation strategies. Attach. Hum. Dev..

[14] Shaver P.R., Mikulincer M. (2002). Attachment-related psychodynamics. Attach. Hum. Dev..

[15] Davis D., Shaver P.R., Vernon M.L. (2003). Physical, emotional, and behavioral reactions to breaking up: The roles of gender, age, environmental involvement, and attachment style. Personal. Soc. Psychol. Bull..

[16] Sbarra D.A. (2006). Predicting the onset of emotional recovery following no marital relationship dissolution: Survival analyses of sadness and anger. Personal. Soc. Psychol. Bull..

[17] Holmberg D., Lomore C.D., Takacs T.A., Price E.L. (2011). Adult attachment styles and stressor severity as moderators of the coping sequence. Pers. Relatsh..

[18] Garrison A.M., Kahn J.H., Miller S.A., Sauer E.M. (2014). Emotional avoidance and rumination as mediators of the relation between adult attachment and emotional disclosure. Personal. Individ. Differ..

[19] Berant E., Mikulincer M., Florian V. (2001). The association of mothers’ attachment style and their psychological reactions to the diagnosis of infant’s congenital heart disease. J. Soc. Clin. Psychol..

[20] Lopez F.G., Gormley B. (2002). Stability and change in adult attachment style over the first-year college transition: Relations to self-confidence, coping, and distress patterns. J. Couns. Psychol..

[21] Messina I., Maniglio R., Spataro P. (2023). Attachment insecurity and depression: The mediating role of interpersonal emotion regulation. Cogn. Ther. Res..

[22] Domic-Siede M., Guzmán-González M., Sánchez-Corzo A., Álvarez X., Araya V., Espinoza C., Zenis K., Marín-Medina J. (2024). Emotion regulation unveiled through the categorical lens of attachment. BMC Psychol..

[23] Lemche E., Giampietro V.P., Surguladze S.A., Amaro E.J., Andrew C.M., Williams S.C., Brammer M.J., Lawrence N., Maier M.A., Russell T.A. (2006). Human attachment security is mediated by the amygdala: Evidence from combined fMRI and psychophysiological measures. Hum. Brain Mapp..

[24] Quirin M., Gillath O., Pruessner J.C., Eggert L.D. (2010). Adult attachment insecurity and hippocampal cell density. Soc. Cogn. Affect. Neurosci..

[25] Eilert D.W., Buchheim A. (2023). Attachment-Related Differences in Emotion Regulation in Adults: A Systematic Review on Attachment Representations. Brain Sci..

[26] Mikulincer M., Shaver P.R. (2023). Attachment Theory Expanded.

[27] Caporael L.R. (2001). Evolutionary psychology: Toward a unifying theory and a hybrid science. Annu. Rev. Psychol..

[28] Sochos A. (2014). Attachment and Social Groups. Attachment Security and the Social World.

[29] Reis H.T., Patrick B.C., Higgins E.T., Kruglanski A.W. (1996). Attachment and intimacy: Component processes. Social Psychology: Handbook of Basic Principles.

[30] Tajfel H., Turner J.C., Austin W.G., Worchel S. (1979). An integrative theory of intergroup conflict. The Social Psychology of Intergroup Relations.

[31] Hogg M.A. (1992). The Social Psychology of Group Cohesiveness: From Attraction to Social Identity.

[32] Prentice D.A., Miller D.T., Lightdale J.R. (1994). Asymmetries in attachments to groups and to their members: Distinguishing between common-identity and common-bond groups. Personal. Soc. Psychol. Bull..

[33] Seeley E.A., Gardner W.L., Pennington G., Gabriel S. (2003). Circle of friends or members of a group? Sex differences in relational and collective attachment to groups. Group Process. Intergroup Relat..

[34] Castano E., Dechesne M. (2005). On defeating death: Group reification and social identification as immortality strategies. Eur. Rev. Soc. Psychol..

[35] Hogg M.A., Sherman D.K., Dierselhuis J., Maitner A.T., Moffitt G. (2007). Uncertainty, entitativity, and group identification. J. Exp. Soc. Psychol..

[36] DeMarco T.C., Newheiser A.K. (2019). Attachment to groups: Relationships with group esteem, self-esteem, and investment in ingroups. Eur. J. Soc. Psychol..

[37] Dekel R., Tuval-Mashiach R. (2012). Multiple losses of social resources following collective trauma: The case of the forced relocation from Gush Katif. Psychol. Trauma Theory Res. Pract. Policy.

[38] Besser A., Neria Y., Haynes M. (2009). Adult attachment, perceived stress, and PTSD among civilians exposed to ongoing terrorist attacks in Southern Israel. Personal. Individ. Differ..

[39] Besser A., Neria Y. (2010). The effects of insecure attachment orientations and perceived social support on posttraumatic stress and depressive symptoms among civilians exposed to the 2009 Israel–Gaza war: A follow-up Cross-Lagged panel design study. J. Res. Personal..

[40] Besser A., Neria Y. (2012). When home isn’t a safe haven: Insecure attachment orientations, perceived social support, and PTSD symptoms among Israeli evacuees under missile threat. Psychol. Trauma Theory Res. Pract. Policy.

[41] Palace M., Zamazii O., Terbeck S., Bokszczanin A., Berezovski T., Gurbisz D., Szwejka L. (2024). Mapping the factors behind ongoing war stress in Ukraine-based young civilian adults. Appl. Psychol. Health Well-Being.

[42] Freh F.M., Cheung Chung M. (2021). Posttraumatic stress disorder and death anxiety among Iraqi civilians exposed to a suicide car bombing: The role of religious coping and attachment. J. Ment. Health.

[43] Tosone C., McTighe J.P., Bauwens J. (2015). Shared traumatic stress among social workers in the aftermath of Hurricane Katrina. Br. J. Soc. Work..

[44] Faul F., Erdfelder E., Buchner A., Lang A.G. (2009). Statistical power analyses using G*Power 3.1: Tests for correlation and regression analyses. Behav. Res. Methods.

[45] Cohen J. (2013). Statistical Power Analysis for the Behavioral Sciences.

[46] Berwick D.M., Murphy J.M., Goldman P.A., Ware J.E., Barsky A.J., Weinstein M.C. (1991). Performance of a five-item mental health screening test. Med. Care.

[47] Wei M., Russell D.W., Mallinckrodt B., Vogel D.L. (2007). The Experiences in Close Relationship Scale (ECR)-short form: Reliability, validity, and factor structure. J. Personal. Assess..

[48] Brennan K.A., Clark C.L., Shaver P.R., Simpson J.A., Rholes W.S. (1998). Self-report measurement of adult attachment: An integrative overview. Attachment Theory and Close Relationships.

[49] Endler N.S., Parker J.D. (1990). Multidimensional assessment of coping: A critical evaluation. J. Pers. Soc. Psychol..

[50] Arbuckle J.L. (2023). Amos (Version 29.0) [Computer Program].

[51] Lynam D.R., Hoyle R.H., Newman J.P. (2006). The perils of partialling: Cautionary tales from aggression and psychopathy. Assessment.

[52] Hair J., Black W.C., Babin B.J., Anderson R.E. (2010). Multivariate Data Analysis.

[53] Byrne B.M. (2010). Structural Equation Modeling with AMOS: Basic Concepts, Applications, and Programming.

[54] George D., Mallery M. (2010). SPSS for Windows Step by Step: A Simple Guide and Reference, 17.0 Update.

[55] Podsakoff P.M., MacKenzie S.B., Lee J.Y., Podsakoff N.P. (2003). Common method biases in behavioral research: A critical review of the literature and recommended remedies. J. Appl. Psychol..

[56] MacKinnon D.P., Fairchild A.J., Fritz M.S. (2007). Mediation analysis. Annu. Rev. Psychol..

[57] Moccia L., Janiri D., Pepe M., Dattoli L., Molinaro M., De Martin V., Chieffo D., Janiri L., Fiorillo A., Sani G. (2020). Affective temperament, attachment style, and the psychological impact of the COVID-19 outbreak: An early report on the Italian general population. Brain Behav. Immun..

[58] Karantzas G., Chesterman S., Ferguson E., Knox L., Lawless N., Mullins E., Romano D., Stokes M.A., Toumbourou J., Westrupp E. (2020). COVID-19 relationship wellbeing & loneliness. PsyArXiv.

[59] Overall N.C., Chang V.T., Pietromonaco P.R., Low R.S., Henderson A.M. (2020). Partners’ attachment insecurity and stress predict poorer relationship functioning during COVID-19 quarantines. Soc. Psychol. Personal. Sci..

[60] Chi X., Becker B., Yu Q., Willeit P., Jiao C., Huang L., Hossain M.M., Grabovac I., Yeung A., Lin J. (2020). Prevalence and psychosocial correlates of mental health outcomes among Chinese college students during the coronavirus disease (COVID-19) pandemic. Front. Psychiatry.

[61] Alfasi Y. (2023). We only know that we don’t know: Attachment patterns and psychological coping during the COVID-19 pandemic–the mediation role of intolerance of uncertainty. J. Soc. Psychol..

[62] Helliwell J.F., Layard R., Sachs J.D., De Neve J.-E., Aknin L.B., Wang S. (2024). World Happiness Report 2024.

[63] Tedeschi R.G., Calhoun L.G. (2004). Posttraumatic growth: Conceptual foundations and empirical evidence. Psychol. Inq..

